# Second hit impels oncogenesis of retinoblastoma in patient-induced pluripotent stem cell-derived retinal organoids: direct evidence for Knudson's theory

**DOI:** 10.1093/pnasnexus/pgac162

**Published:** 2022-08-17

**Authors:** Yan-Ping Li, Ya-Ting Wang, Wen Wang, Xiao Zhang, Ren-Juan Shen, Kangxin Jin, Li-Wen Jin, Zi-Bing Jin

**Affiliations:** Beijing Institute of Ophthalmology, Beijing Tongren Hospital, Capital Medical University, Beijing 100730, China; Beijing Institute of Ophthalmology, Beijing Tongren Hospital, Capital Medical University, Beijing 100730, China; Beijing Institute of Ophthalmology, Beijing Tongren Hospital, Capital Medical University, Beijing 100730, China; Beijing Institute of Ophthalmology, Beijing Tongren Hospital, Capital Medical University, Beijing 100730, China; Beijing Institute of Ophthalmology, Beijing Tongren Hospital, Capital Medical University, Beijing 100730, China; Beijing Institute of Ophthalmology, Beijing Tongren Hospital, Capital Medical University, Beijing 100730, China; Quanzhou Aier Eye Hospital, Quanzhou 362017, China; Beijing Institute of Ophthalmology, Beijing Tongren Hospital, Capital Medical University, Beijing 100730, China

**Keywords:** retinoblastoma, iPS cells, retinal organoids, two-mutation hypothesis, RB1

## Abstract

Retinoblastoma (Rb) is a type of malignant tumor due to abnormal retinogenesis with biallelic mutations of the *RB1* gene. Its pathogenesis has been proposed as a “two-mutation hypothesis” by Knudson since 1971; however, there remain some debates on disease onset sufficiency of the biallelic *RB1* mutations. To obtain straightforward evidence for this hypothesis, we investigated whether two-hit mutations of the *RB1* gene drive tumorigenesis in patient-induced pluripotent stem cell (hiPSC)-derived human retinal organoids (hROs) and whether single allelic mutation hiPSC-derived hROs exhibit molecular and cellular defects. We generated hiPSCs with a heterozygous germline mutation (*RB1^m1/^^wt^*) from a Rb patient. A second-allele *RB1* gene mutation was knocked in to produce compound heterozygous mutations (*RB1^m1/m2^*) in the hiPSCs. These two hiPSC lines were independently developed into hROs through a stepwise differentiation. The hiPSC-*RB1^m1/m2^* derived organoids demonstrated tumorigenesis in dishes, consistent with Rb profiles in spatiotemporal transcriptomes, in which developmentally photoreceptor fate-determining markers, CRX and OTX2, were highly expressed in hiPSC-*RB1^m1/m2^* derived hROs. Additionally, ARR3^+^ maturing cone precursors were co-labeled with proliferative markers Ki67 or PCNA, in agreement with the consensus that human Rb is originated from maturing cone precursors. Finally, we demonstrated that retinal cells of hROs with monoallelic *RB1* mutation were abnormal in molecular aspects due to its haploinsufficiency. In conclusion, this study provides straightforward supporting evidence in a way of reverse genetics for “two-hit hypothesis” in the Rb tumorigenesis and opens new avenues for development of early intervention and treatment of Rb.

Significance Statement According to Knudson's “two-hit” hypothesis, biallelic *RB1* mutations are critical for retinoblastoma (Rb) formation, but there is no biological model to test this hypothesis in a straightforward way. Here, we constructed Rb organoids derived from patient-induced pluripotent stem cells (patient-iPSCs) with a germline “hit” in the *RB1* gene and an extra-mutation knocked in by CRISPR/Cas9. Under stepwise differentiation, the Rb organoids were successfully formed in Petri dishes with typic tumor signatures and excessive proliferation of cone precursors. In addition, although the patient-iPSCs with a monoallelic mutation did not produce Rb, significant alterations in molecular aspects presented in the retinal organoids due to the haploinsufficiency. In brief, we established an Rb organoid model with patient-specific genetic background and provided straightforward evidence testifying Knudson's theory.

## Introduction

Retinoblastoma (Rb) is the most commonly intraocular malignant tumor affecting young children and can be lethal if left untreated. Rb, developed in an infant, was usually initiated by *RB1* biallelic mutations (1). In 1971, based on mathematical models, Alfred G. Knudson put forward the “two-hit” hypothesis for Rb formation (2). Heritable Rb is usually caused by the inheritance of a germline mutation (“first hit, M1”) and followed by a somatic mutation in the second allele (“second hit, M2”) (2, 3). In nonheritable Rb, instead of occurrence in germline cells, two hits occur in the same susceptible somatic cell during retinogenesis (4). Later, Knudson's theory was advanced from genetic studies in which the recessive nature of the gene mutation was validated (5). Additionally, several researchers analyzed clinical data and found that genetics of Rb patients followed Knudson's curves (6, 7), which supports the two-hit theory. However, apart from clinical data and mathematical models (8), there is no available biological model of Rb development that could verify Knudson's hypothesis. Genomic studies have demonstrated that biallelic mutations in the*RB1* gene are sufficient for oncogenesis of Rb (9). Similarly, studies in mouse models showed that inactivation of *Rb1* leads to embryonic death by E14.5 (10–12), and Rb only develops when the *Rb1* gene is lost together with inactivation of other Rb-related genes during retinogenesis (13). These models are far from clinical relevance to the inactivation of the *RB1* gene in human Rb.

As an inspiration of oncogenesis for the “two-hit” hypothesis, inactivation of both *RB1* alleles is necessary for initiating Rb (14). Recently, a line of evidence shows that *RB1* homozygous mutation possibly leads to benign retinoma formation, and additional genetic initiatings are needed for malignant transformation (15–17), against the two-mutation hypothesis of Rb. Although several cytogenetic and comparative genomic hybridization studies were used to show the insufficiency of *RB1* inactivation for determining Rb, these are all based on the consequences of solid tumor, and they could not preclude that loss of *RB1* function leads to genomic instability and even more “hits” (15, 16, 18). Additionally, retinomas are rarely reported in humans, only present in about 0.7% of carriers with germline *RB1* mutation (19), and there has been no documentation of the equivalent retinomas in other mammalian animal models. These studies either supported or denied the Knudson hypothesis, which warranted further studies of direct evidence in support or opposition of the hypothesis.

Pluripotent stem cells (PSCs), including embryonic stem cells (ESCs) and induced PSCs (iPSCs), can self-renew and be stably maintained in vitro. PSCs are capable of differentiating into all germ layers, and also can mirror organogenesis under specific differentiation protocols in vitro (20). Combined with effective gene-editing technology, PSCs can be genetically manipulated to contract disease-causative mutations (21).

Organoids, the recent development of 3D culture technology, provide a completely new platform for studying tumorigenesis (22). To a certain extent, organoids can recapitulate organ morphogenesis in cellular hierarchy and dynamics, and present the cellular and genetic heterogeneity of native tissues in vitro (23). In like manner, human retinal organoids (hROs) can be well established from hESCs or hiPSCs with gradual adaptation of protocols (20, 24–26). The self-organized hROs contain different retinal cell types in a polarized and laminated structure, in which photoreceptor cells have electrophysiological properties and light-evoked responses (27–30). Rb is a well-established model to study tumorigenesis due to a single gene inactivation (1). The depletion of *RB1* from hESCs established a successful Rb model. Loss of *RB1* in hROs fully recapitulated Rb formation process in differentiation, phenotype, and function. Besides, upon dissemination of cells from Rb-containing organoids (RBOs) into immune-deficient mice, tumors did successfully be generated in the host, verified by phenotypes and marker genes (31).

Although hESCs-derived tumor organoids can reproduce the tumorigenesis process, this model lacks the patient-specific genetic background and is not an optimal preclinical model for precise medicine. Recently, Norrie et al. generated hRBOs derived from patient-specific iPSCs with germline *RB1* mutations. After the dissociation and injection of hRO cells in the vitreous cavity of immune-compromised mice, RBOs were formed with molecular, cellular, and genomic features of human Rb (32). This model carries patient's genetic information, but there are various uncontrollable factors in the process of tumorigenesis, and as a result, the tumor development occurs stochastically. Additionally, in immunocompromised mice, the microenvironment of Rb is very different from that of the patient, and the tumorigenic potential of Rb may be different from Knudson's two-hit theory. Thus, disease models should more closely or precisely represent tumor progression. The development of hROs has enabled the precise customization of new models of tumorigenesis, and they may serve as a promising alternative for in vivo studies. In this study, we provide direct evidence of reverse genetics with patient-specific iPSCs testifying the two-hit hypothesis of the *RB1* gene inactivation. Importantly, our Rb model offers a unique opportunity to probe the behaviors of Rb and to examine the consequences of two-hit in tumorigenesis.

## Results

### Generation, modification, and characterization of Rb patient-derived iPSCs

**Fig. 1.**
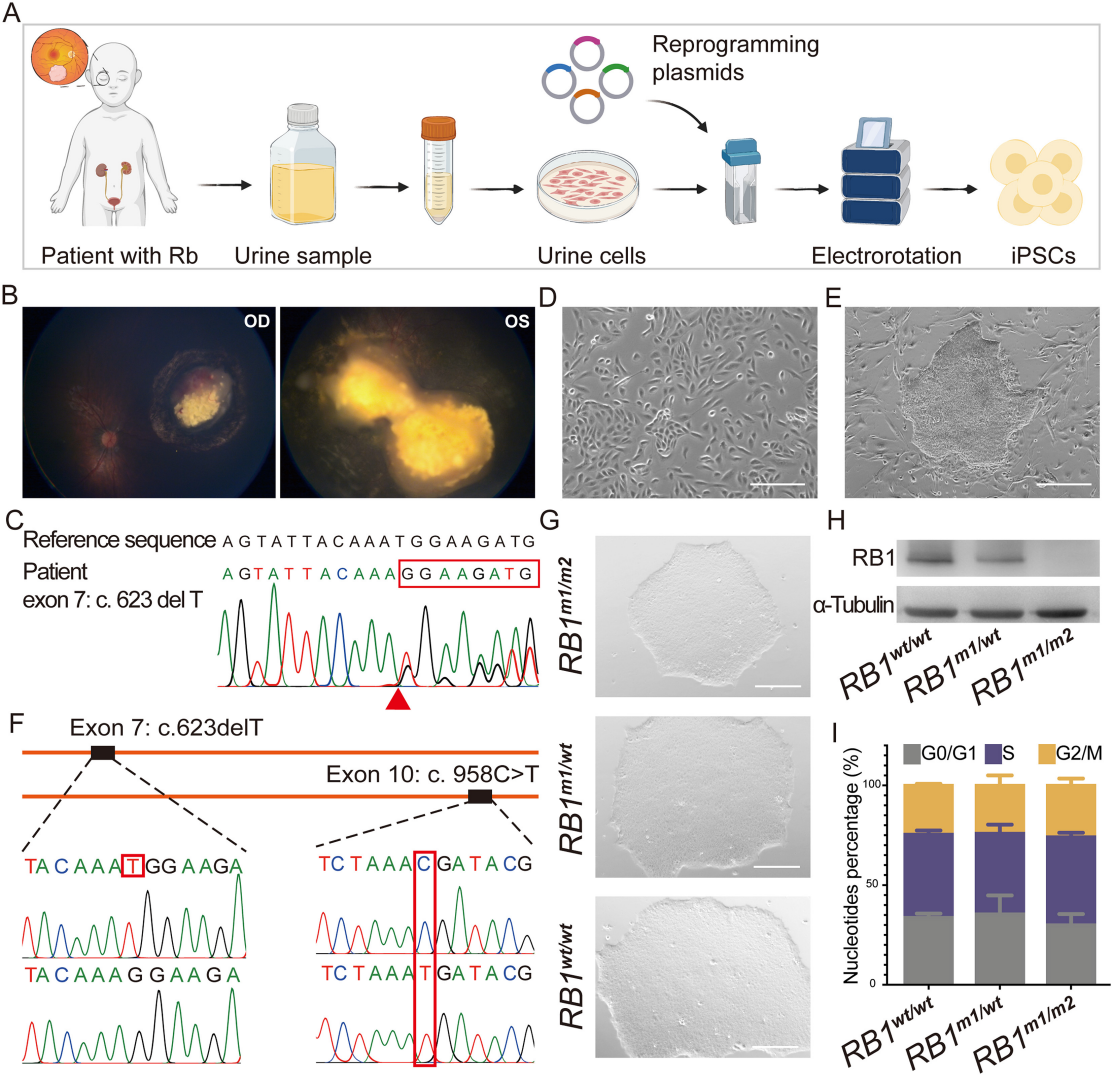
Generation, modification, and characterization of Rb patient-derived iPSC lines. (A) Scheme of hiPSC establishment from urine sample of the Rb patient. (B) Fundus appearances of the bilateral Rb after therapy. OD, right eye. OS, left eye. (C) Sanger sequencing of the *RB1* gene in the Rb patient. A heterozygous 1-bp deletion in exon 7 results in a frameshift mutation (c.623delT, p.M208fs). (D) Representative bright filed image of urine cells. Scale bars, 200 µm. (E) Representative bright filed image of a hiPSC colony. Scale bars, 400 µm. (F) Sanger sequencing of cDNA sequence of targeted loci in iPSC-*RB1^m1/m2^*. (G) Representative bright filed images of iPSC-*RB1^wt/^^wt^*, -*RB1^m1/^^wt^*, and -*RB1^m1/m2^*colonies. Scale bars, 400 µm. (H) Western blot analysis for RB1 protein expression in iPSC-*RB1^wt/^^wt^*, -*RB1^m1/^^wt^*, and -*RB1^m1/m2^* cells. (I) Relative percentage of cells within each cell cycle phase of iPSC-*RB1^wt/^^wt^*, -*RB1^m1/^^wt^*, and -*RB1^m1/m2^* lines. *n* = 3 for all samples.

A 10-month-old boy was detected to have white reflex in his left eye during routine pediatric examination. Grading of initial tumors showed that the tumor affecting the right eye was in Group C and that of left eye was in Group E according to the International Classification for Intraocular Retinoblastoma (IIRC). After the patient was given three cycles of intra-arterial chemotherapy, four cycles of primary systemic chemotherapy, and laser therapy in 3 months, fundus examination revealed a huge tumor involved the optic disc in the left fundus (Fig. 1B) and a yellow-white lesion with multiple areas of calcification in the upper nasal quadrant of right eye. Sanger sequencing of DNA extracted from the patient peripheral blood identified a heterozygous frameshift mutation (exon7: c.623delT; p.M208fs) in the *RB1* gene (NM_000321) (Fig. 1C). For the benefit of patient, the tumor tissues were directly eliminated with LASER operations and not available for searching the second mutation in the *RB1* gene. While the second mutation was not identified, we supposed that it might be present in somatic tumor cells as previously reported (2, 33). Urinary cells were isolated from 50 to 100 mL patient urine samples, and these cells displayed spindle-shaped morphology (Fig. 1D). The iPSC-*RB1^m1/^^wt^* colonies derived from the patient urinary cells had typical PSC clone morphology and cells in colonies displayed a high nuclei/cytoplasm ratio and were tightly packed (Fig. 1E).

In exon 10 of *RB1*, the c. 958C > T (p.Arg320X) introduces a nonsense mutation. It involves a C to T conversion at codon 320, and changes the CGA-arginine codon to stop codon (TGA). To generate biallelic mutations in the *RB1* gene, we knocked-in a c. 958C > T mutation in the wildtype allele of *RB1* in iPSC-*RB1^m1/^^wt^* by targeting exon 10 of the *RB1* gene using the CRISPR/Cas9 gene editing system (Fig. 1F; Fig. S1A). By Sanger sequencing, we verified that the edited iPSC line had the proposed mutation (exon10: c. 958C > T) in the *RB1* gene (Fig. 1F). Both the iPSC-*RB1^m1/^^wt^* and iPSC-*RB1^m1/m2^* lines showed the same karyotype as the iPSC-*RB1^wt/^^wt^* (Fig. S1B) and maintained a classical tightly packaged dome-shaped PSC morphology (Fig. 1G). Western blotting and immunofluorescence staining results suggested that *RB1* expression is lower in the iPSC-*RB1^m1/^^wt^* than that in iPSC-*RB1^Wt/^^Wt^*, but not detectable in iPSC-*RB1^m1/m2^* (Fig. 1H; Fig. S1C), in accordance with the expected protein dosages.

As a tumor suppressor gene, *RB1* has a key role in regulating cell cycle progression and cell differentiation. Retinoblastoma protein (RB1), encoded by the *RB1* gene, interacts with SKP2 and E2F1 to inhibit cell cycle progression and blocks G1-S phase transition (34). To investigate the effect of *RB1* mutations in heterozygous and compound heterozygous hiPSC lines, the cell cycle properties were evaluated by flow cytometry. Compared with wild type (hiPSC-*RB1^wt/^^wt^*), the cell cycle was not altered in two lines (Fig. 1I; Fig. S1D). Additionally, RNA sequencing (RNA-seq) analysis verified that the expression of cell cycle and pluripotency-related genes was not affected in either heterozygous or compound heterozygous hiPSC line (Fig. S1E).

Immunofluorescence staining of iPSC-*RB1^m1/^^wt^* and iPSC-*RB1^m1/m2^* confirmed that the expression of several key pluripotency protein markers, including SOX2, SSEA4, NANOG, and OCT4 (Fig. S1F), was unaffected. Alkaline phosphatase (AP) staining of iPSCs demonstrated a high expression of AP in all iPSCs lines (Fig. S1G). These iPSCs could differentiate into cell types of three germ layers through embryoid bodies (EBs)-mediated differentiation in vitro. Immunofluorescence staining showed that some differentiated cells were positive for AFP (endoderm marker), ∂-SMA (mesoderm marker), or GFAP (ectoderm marker) (Fig. S1G), demonstrating the pluripotency of the cell lines.

## Tumor differentiation in hiPSCs-derived hROs with *RB1* compound heterozygous mutations

**Fig. 2.**
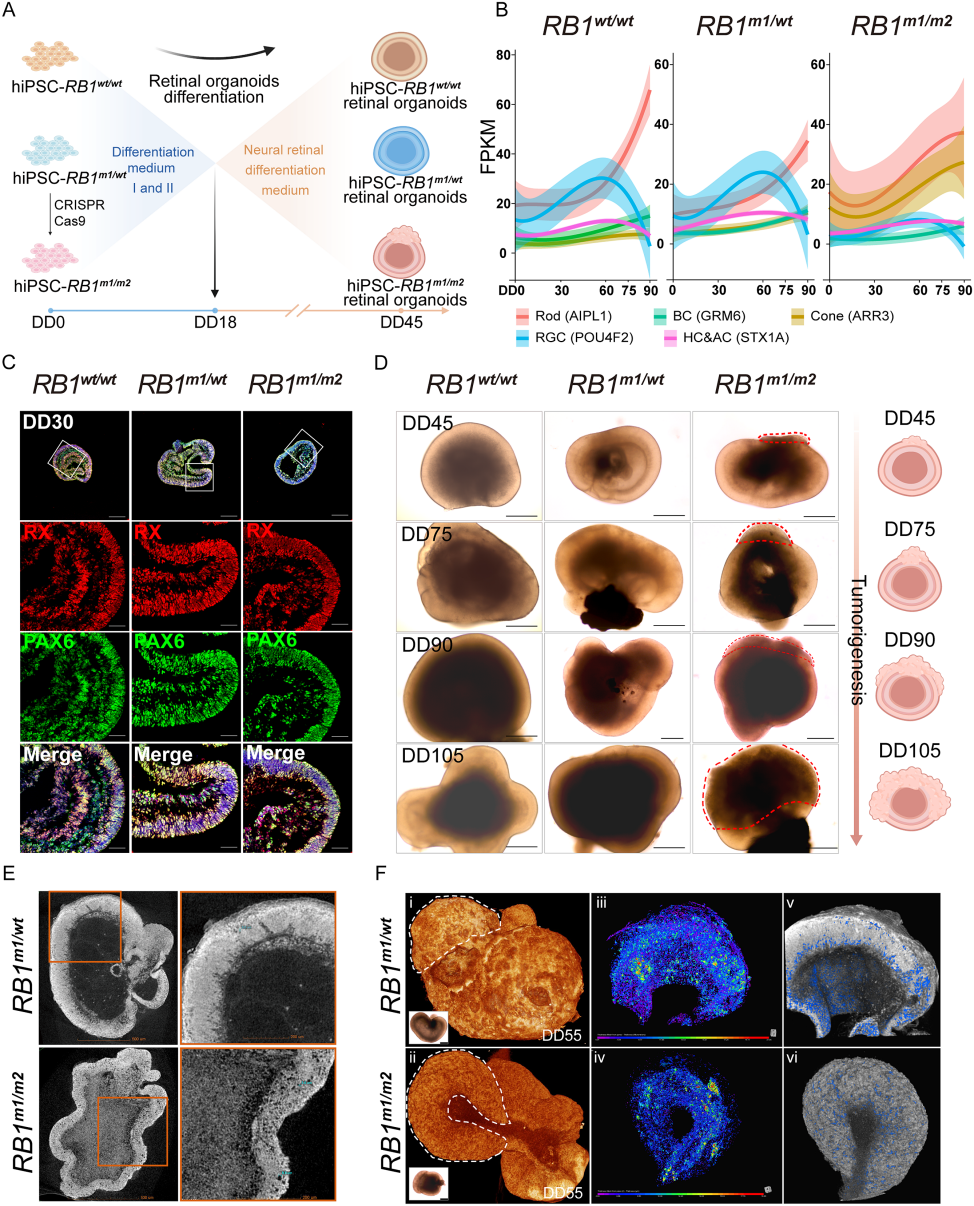
Characterization of hROs derived from iPSC-*RB1^wt/^^wt^*, -*RB1^m1/^^wt^*, and -*RB1^m1/m2^*. (A) Scheme of hROs differentiation derived from iPSC-*RB1^wt/^^wt^*, -*RB1^m1/^^wt^*, and -*RB1^m1/m2^*. DD, differentiation day. (B) Retinal cell genesis at different timepoints in hROs. Each curve represented the FPKM of representative gene for each retinal cell over the time in hROs. AIPL1 for rod; ARR3 for cone; GRM6 for bipolar cell (BC); POU4F2 for RGC; and STX1A for horizontal cell and amacrine cell (HC and AC). *n* = 3. (C) Immunostaining of retinal progenitor cell markers RX and PAX6 in three groups of hROs at DD30. The top panel shows representative images of each group. Scale bars in top panel, 200 µm. The boxed regions in top panel were magnified in the middle and bottom panels accordingly. Scale bars in middle and bottom panels, 50 µm. Cell nucleus was counterstained with DAPI (blue). (D) Representative images of hROs (or hRBOs) at different timepoints. Right schematic diagrams showed the process of tumorigenesis in *RB1^m1/m2^* hRBOs. Red dashed lines delineated the borders of tumors in *RB1^m1/m2^* hRBOs. Scale bars, 400 µm. (E) The 2D virtual slice of micro-CT. The boxed regions were magnified in the right. (F) The micro-CT images of hROs and hRBOs at DD55. (i) The reconstructed 3D image of the *RB1^m1/^^wt^* hRO scanned with 1.5 µm vortex resolution. The bright-filed image of *RB1^m1/^^wt^* hRO was shown in the bottom left corner, Scale bars, 400 µm. (ii) The reconstructed 3D image of the *RB1^m1/m2^* hRBO scanned with 1.2 µm vortex resolution. The bright-filed image of *RB1^m1/m2^* hRBO was shown in the bottom left corner, Scale bars, 400 µm. White dashed lines in (i) and (ii) were used to enclose the regions for porosity calculation. (iii to vi) The 3D rendering images were obtained from Dragonfly software's thickness analysis of the organoid's surface and internal pores (indicated in blue).

To investigate in vitro tumorigenesis of Rb with patient-specific genomic background, we generated hROs from iPSC-*RB1^m1/^^wt^* and iPSC-*RB1^m1/m2^* using stepwise induction as described previously with slight modification (27, 31, 35, 36) (Fig. 2A). By RNA-seq analysis of iPSC-*RB1^m1/^^wt^* derived hROs during retinogenesis, we found that retinal ganglion cells (RGCs) markers appeared first, followed by those of horizontal and amacrine cells and finally those of photoreceptor and bipolar cells. The result demonstrated that the iPSC lines and method we used are capable of recapitulating the genesis of the whole repertoire of retinal cells in the same chronological order in vivo (Fig. 2B; Fig. S2A). The in vitro morphogenetic and molecular properties also replicated retinogenesis. In the early stage of differentiation (differentiation day 30, DD30), in terms of the morphology of ROs and the expression of eye field related proteins, there were no differences among iPSC-*RB1^wt/^^wt^*, -*RB1^m1/^^wt^*, and -*RB1^m1/m2^* derived hROs (Fig. 2C; Fig. S2B and C). At the later stage of differentiation (DD90), the expression of cone-arrestin in iPSCs-*RB1^m1/m2^* derived hRBOs was much higher than that in other two groups (Fig. 2B).

However, starting from DD45 and beyond, the neoplasm, similar to tumor primary foci, began to appear in the ROs derived from iPSC-*RB1^m1/m2^* but not -*RB1^wt/^^wt^* or -*RB1^m1/^^wt^*, which disrupted the original structure of neural retina and bulged outward (Fig. 2D; Movies S1). The tumor grew rapidly and its edge was irregular (Fig. 2D; Movies S1). To further characterize the neoplasm, high-resolution X-ray computer tomography (micro-CT) scanning was used to examine the internal laminar structure and cellular arrangement of ROs (Fig. S2D). Rosette-like structure is one of the pathological hallmarks of differentiated Rb (37), and Liu et al. found that *RB1*-depleted hRBOs had structures of Flexner–Wintersteiner rosettes and fleurettes (31), which resulted in a sparse Rb structure containing micron-sized pores in the rosettes. The neural retina and the internal pores can be clearly seen on the 2D virtual slice of micro-CT, and there were more pores in *RB1^m1/m2^* hROs but barely seen in *RB1^m1/^^wt^* hROs (Fig. 2E). Besides, it also can be seen from the 3D rendering images reconstructed by micro-CT that the morphology of *RB1^m1/m2^* hROs is more irregular than that of the other two groups (Fig. 2F(i) and (ii); Fig. S2E(i) and (ii); Movies S2 to S4). And it also showed that *RB1^m1/^^wt^* hROs had more compact outer tissue (Fig. 2F). The porosity was computed based on the approximate volume of 3D tomogram and was 3%, 5.1%, and 6.1% in the hROs of *RB1^wt/^^wt^, RB1^m1/^^wt^*, and *RB1^m1/m2^* [Fig. 2F(iii) to (vi); Fig. S2E(iii) and (iv)], respectively, indicating a gradual increase in the ratio of pores in the single and compound heterozygous hROs. These results demonstrated that *RB1* mutation(s) disrupted cellular and tissue structures in retinas.

### Molecular profiling of hiPSCs derived tumors with compound heterozygous mutations

**Fig. 3.**
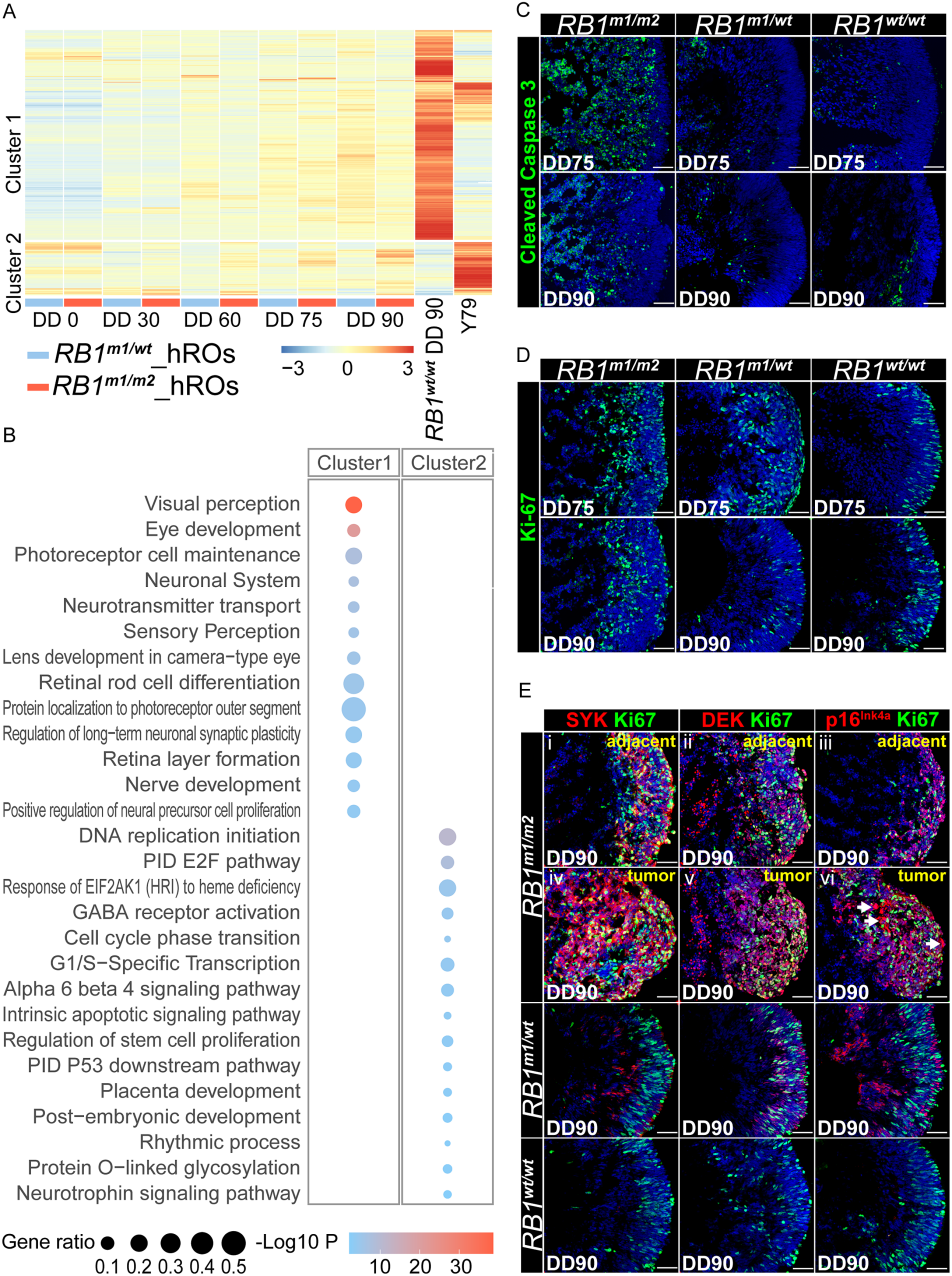
Molecular profiling and characterization of hROs derived from iPSC-*RB1^wt/^^wt^*, -*RB1^m1/^^wt^*, and -*RB1^m1/m2^*. (A) Heatmap showed the DEGs between *RB1^m1/^^wt^* and *RB1^m1/m2^* hROs at various differentiation timepoints. Hierarchical clustering of the significantly expressed genes identified two main gene clusters. Cluster 1: gene expression levels of 419 upregulated genes in *RB1^m1/^^wt^* hROs. Cluster 2: gene expression levels of 106 upregulated genes in *RB1^m1/m2^* hRBOs. Light blue, *RB1^m1/^^wt^* hROs, *n* = 3. Red, *RB1^m1/m2^* hRBOs, *n* = 3. DD, differentiation day. Control, *RB1^wt/^^wt^* hROs, *n* = 3. Y79, human Rb cell line, *n* = 4. (B) Significant gene sets from the Metascape enriched in clusters 1 and 2. (C) Immunostaining of cleaved caspase 3 in hROs and hRBOs. Representative images were shown for each group at DD75 and DD90. (D) Representative immunostaining images of Ki67 were shown for each group at DD75 and DD90. (E) Immunostaining of SYK, DEK, p16^Ink4a^ and Ki67 in hROs and hRBOs at DD90. (i to iii) Representative images of the adjacent tissues in hRBOs; (iv to vi) Representative images of the solid tumors in hRBOs. White arrows pointed to cells with high cytoplasmic p16^Ink4a^ in hRBOs. Scale bars, 50 µm. Cell nucleus was counterstained with DAPI (blue in C to E).

To further identify the molecular characteristics of Rb-containing retinal organoids (hRBOs), RNA-seq analyses of hROs from iPSC-*RB1^wt/^^wt^*, -*RB1^m1/^^wt^*, and -*RB1^m1/m2^* were performed. We identified the differentially expressed genes (DEGs) between *RB1^wt^^/^^wt^* at DD90 and human Rb cell line Y79, and projected them to each differentiation timepoints. A heat-map with two major clusters of the DEGs between *RB1^m1/^^wt^* and *RB1^m1/m2^* was displayed (Fig. 3A). A total of 419 genes were upregulated in *RB1^m1/^^wt^* hROs and 106 genes were upregulated in *RB1^m1/m2^* hRBOs (Fig. 3A). To unravel the potential mechanism in these gene list, we performed gene enrichment analysis in Metascape and found that the significant ontology terms in cluster 1 were related to retinogenesis and maturation, such as eye development and retinal rod cell differentiation. In cluster 2, the significant ontology terms were related to cell cycle, such as DNA replication pathway and G1/S-specific transcription (Fig. 3B). To assess the apoptotic cell death, immunostaining for cleaved caspase 3 was performed. We observed that cleaved caspase 3 was more abundant in *RB1^m1/m2^* hRBOs (Fig. 3C; Fig. S3A and C). Consistent with the notion that cell proliferation is the main characteristic of Rb and other tumors, we found higher ratio of Ki67^+^ cells in *RB1^m1/m2^* hRBOs (Fig. 3D; Fig. S3A and C). Compared with *RB1^m1/^^wt^* or *RB1^wt/^^wt^* hROs, Rb-related markers SYK and DEK were both highly expressed in the solid tumor and the adjacent tissues in *RB1^m1/m2^* hRBOs by immunostaining (Fig. 3E; Fig. S3B and D).

Clinically, Rb was developed from retinoma stage (38–40), and in most cases, retinoma often coexisted with Rb (15). We explored whether *RB1^m1/m2^* hRBOs had retinoma-associated features. Indeed, immunostaining of retinoma-related p16^INK4a^ (41) suggested that retinoma was mixed with Rb lesion and had strong cytoplasmic staining for p16^INK4a^ (p16^INK4a_hi^) but lacked Ki67 at DD90 and DD105 (Fig. 3E; Fig. S3D). And the cells with Ki67 as well as a low level of cytoplasmic p16^INK4a^ (p16^INK^) were commonly found in Rb but not in retinoma (Fig. 3E; Fig. S3D). These results showed the presence of many Rb cells in *RB1^m1/m2^* hRBOs and provided evidence that biallelic mutations of *RB1* can cause Rb tumorigenesis during retinogenesis in retinal organoids.

### Cone precursor is the cell-of-origin of retinoblastoma

**Fig. 4.**
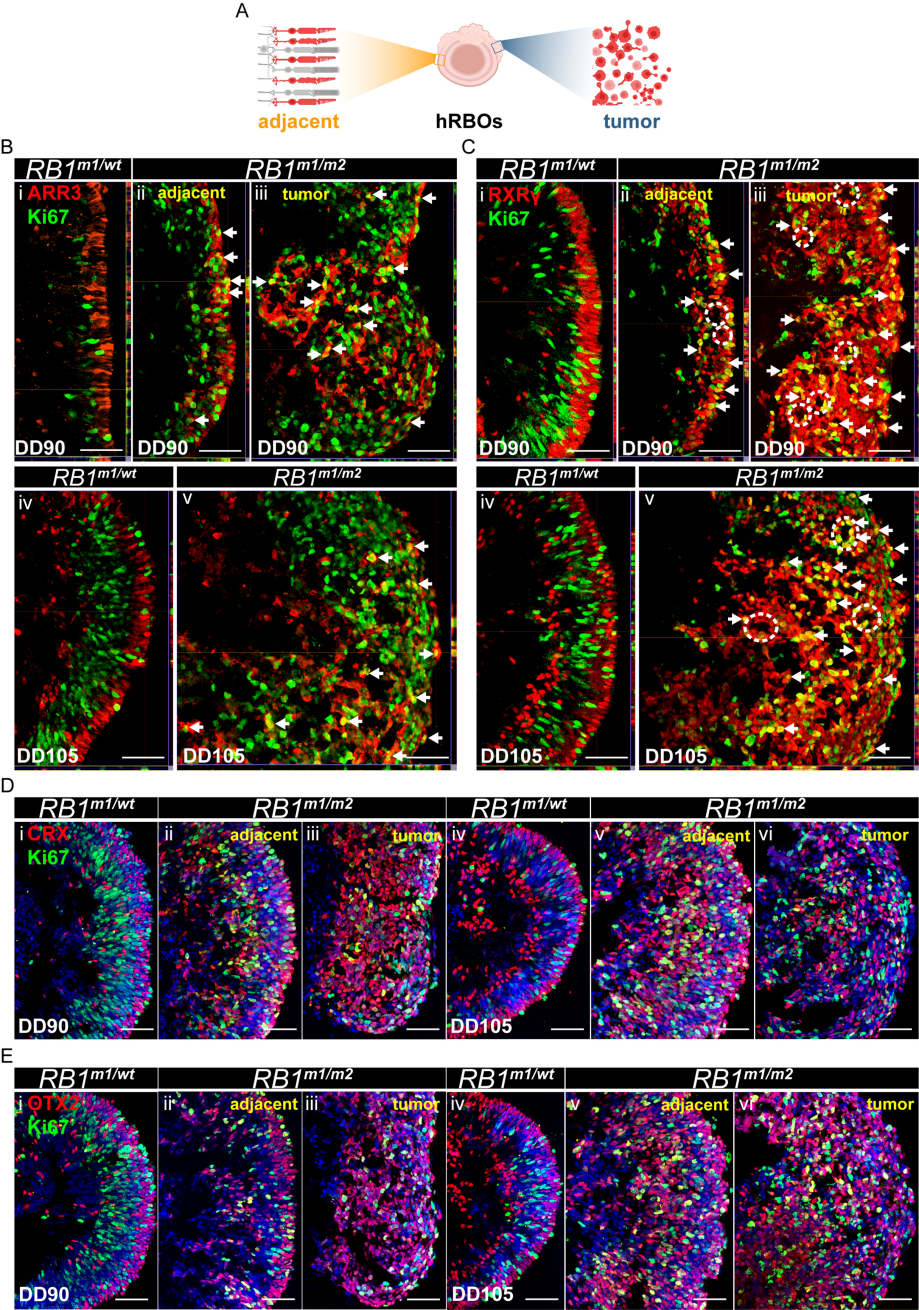
Rb tumor and adjacent tissues highly express cone and cone precursor markers. (A) Top image illustrates the locations of tumors and adjacent tissues. (B and C) Immunostaining of Ki67 and maturing cone marker ARR3 (in B) or cone precursor marker RXRγ (in C) in *RB1^m1/^^wt^* hROs and *RB1^m1/m2^* hRBOs at DD90 and 105. Representative images were shown for the (i) *RB1^m1/^^wt^* hROs at DD90, (ii) adjacent tissue in *RB1^m1/m2^*hRBOs at DD90, (iii) tumor in *RB1^m1/m2^* hRBOs at DD90, (iv) *RB1^m1/^^wt^* hROs at DD105, and (v) *RB1^m1/m2^* hRBOs at DD105. White arrows pointed to cells with co-expression of Ki67 with ARR3 or RXRγ. Dashed circles enclosed the Flexner–Wintersteiner rosette-like structures. Scale bars, 50 µm. (D and E) Immunostaining of Ki67 and photoreceptor transcription factors CRX (in D) or OTX2 (in E) in *RB1^m1/^^wt^* hROs and *RB1^m1/m2^* hRBOs at DD90 and DD105. Representative images were shown for the (i) *RB1^m1/^^wt^* hROs at DD90, (ii) adjacent tissue in *RB1^m1/m2^*hRBOs at DD90, (iii) tumor in *RB1^m1/m2^*hRBOs at DD90, (iv) *RB1^m1/^^wt^* hROs at DD105, (v) adjacent tissue in *RB1^m1/m2^* hRBOs at DD105, and (vi) tumor in *RB1^m1/m2^* hRBOs at DD105. Cell nucleus was counterstained with DAPI (blue). Scale bars, 50 µm.

In Rb cells, it has been reported that cone precursor cells are sensitive to *RB1* depletion (41, 42). And our recent study supported that Rb is originated from maturing cone precursors (31). To confirm that the proliferating Rb cells in hRBOs are derived from postmitotic cone precursors, we examined Rb cells in *RB1*-deficient hRBOs. Immunostaining revealed that the proliferating cone-like cells (ARR3^+^ Ki67^+^) were increased in the adjacent tissue and the solid tumor of hRBOs compared to hROs (Fig. 4B; Fig. S4A). Compared with *RB1^m1/^^wt^* hROs, immunostaining showed that cone precursor marker RXRγ was more expressed in the solid tumor and the adjacent tissues in *RB1^m1/m2^* hRBOs (Fig. 4C; Fig. S4A). As indicated by immunostaining, the hRBOs also have abundant Flexner–Wintersteiner rosette structures (Fig. 4C; Fig. S4A, white circles), which is a typical feature in Rb tissues. Besides, the tumor in hRBOs highly expressed the photoreceptor markers CRX and OTX2, in agreement with Rb being derived from cone-committed cells (Fig. 4D and E; Fig. S4B). PCNA, a marker of proliferating cell, was extensively co-labeled with ARR3 in *RB1^m1/m2^* hRBOs at DD90, whereas this co-labeling was rarely observed in other two groups of hROs (Fig. S4C). For verification at DD120, most of ARR3^+^, CRX^+^, or OTX2^+^ cells were colocalized with proliferative marker PCNA or Ki67 (Fig. S4D). We also found that the Rb marker SYK and ARR3 were co-expressed in many cells of hRBOs (Fig. S4E). Taken together, these results endorsed our and others’ previous finding that cone precursors were the original source of Rb.

### Focal retinal dysplasia in *RB1* heterozygous hROs

**Fig. 5.**
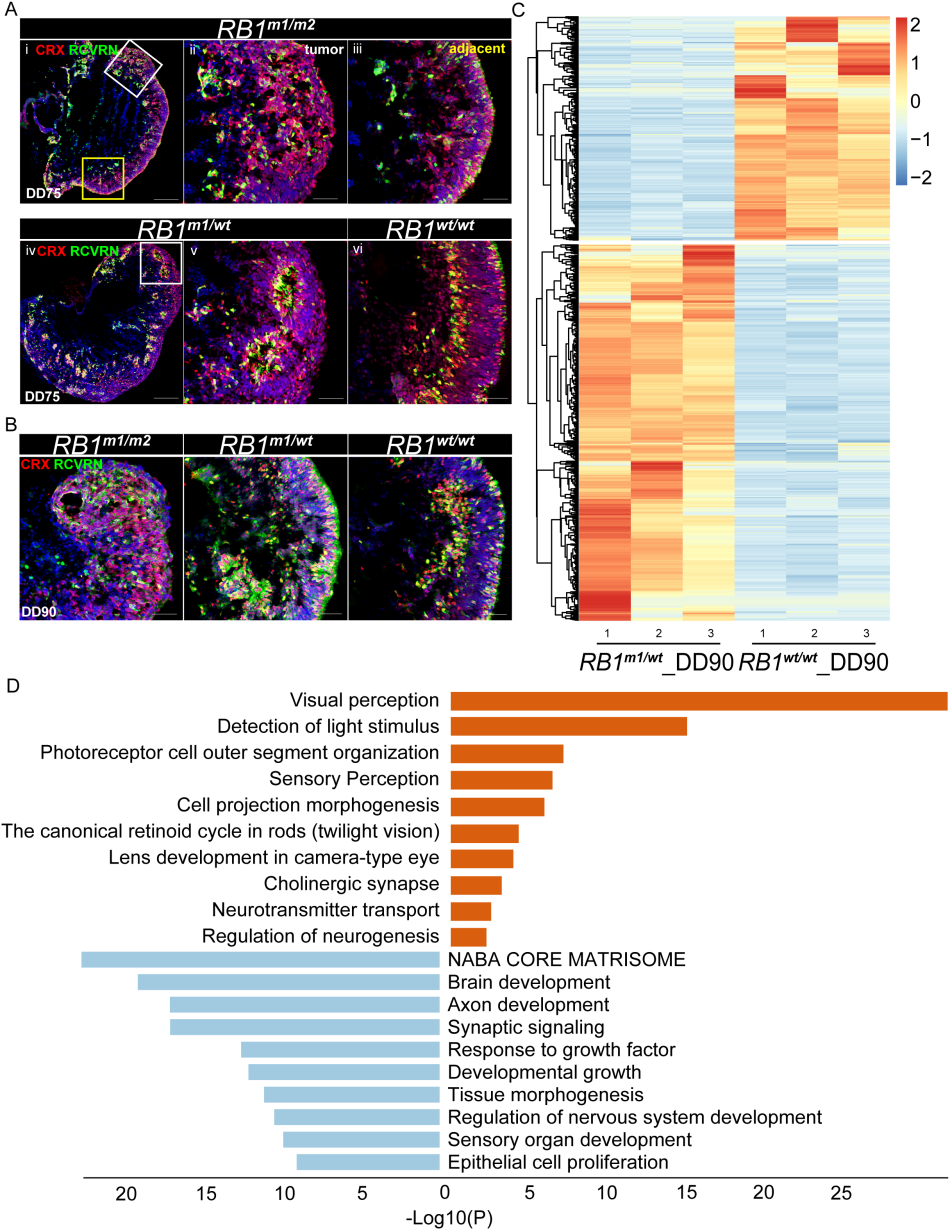
*RB1* heterozygous retinal cells in hROs have developmental defects and are inherently different from wildtype ones. (A) Representative images of immunostaining of photoreceptor markers CRX and RCVRN in *RB1^m1/m2^* hRBOs, *RB1^m1/^^wt^* hROs, and *RB1^wt/^^wt^* hROs at DD75. The white-boxed region and yellow-boxed region in (i) were magnified in (ii) or (iii), respectively, which represented the tumor or the adjacent tissue in hRBOs, respectively. The white-boxed region in (iv) was magnified in (v), showing the region with typical retinal dysplasia in *RB1^m1/^^wt^* hROs but not in *RB1^wt/^^wt^* hROs. Cell nucleus was counterstained with DAPI (blue). Scale bars, 100 µm (i and iv), and 50 µm (ii, iii, v, and vi). (B) Representative images of immunostaining of photoreceptor markers CRX and RCVRN in *RB1^m1/m2^* hRBOs, *RB1^m1/^^wt^* hROs, and *RB1^wt/^^wt^* hROs at DD90. Cell nucleus was counterstained with DAPI (blue). Scale bars, 50 µm. (C) Heatmap showed the DEGs in hROs between *RB1^m1/^^wt^* and *RB1^wt/^^wt^* at DD90. *n* = 3. (D) Enrichment of the significant gene sets in hROs between *RB1^m1/^^wt^* and *RB1^wt/^^wt^* by Metascape. Orange and light blue colors represented enriched gene sets in *RB1^wt/^^wt^* hROs and *RB1^m1/^^wt^* hROs, respectively.

An interesting question is that whether retinal cells with one allele of *RB1* inactivation are normal or have defects caused by haploinsufficiency of *RB1*. To address the question, we investigated whether *RB1^m1/^^wt^* hROs exhibited any developmental defects. Immunostaining of *RB1^m1/^^wt^* hROs at DD75 and DD90 confirmed the presence of minor focal retinal dysplasia (Fig. 5A and B). In addition, it also showed that photoreceptors (CRX^+^ and RCVRN^+^) were present in most dysplasia (Fig. 5A and B), implying that these photoreceptors could be abnormal. We performed RNA-seq analysis of *RB1^wt/^^wt^* and *RB1^m1/^^wt^* hROs at DD90 to examine the underlying molecular alterations. We found a comprehensive change in the profiling of DEGs between *RB1^m1/^^wt^* and *RB1^wt/^^wt^* hROs (Fig. 5C). A total of 419 genes were upregulated in *RB1^wt^^/^^wt^* hROs and 704 genes were upregulated in *RB1^m1/^^wt^* hROs (Fig. 5C). To unravel the potential pathways and mechanism, we performed gene enrichment analysis in Metascape and found that genes highly expressed in *RB1^wt/^^wt^* hROs are enriched in retinal development and maturation (Fig. 5D). Surprisingly, the significant ontology terms of highly expressed genes in *RB1^m1/^^wt^* hROs were enriched in brain development, axon development and synaptic signaling (Fig. 5D), indicating an indispensable role of *RB1* in normal neural development. The data suggest that retinal cells with one functional copy of *RB1* had developmental disorders during retinogenesis.

## Discussion

Given the poor prognosis of Rb, there is an urgent need to establish an in vitro Rb model to better recapitulate human Rb development, uncover the pathological mechanism of tumorigenesis, trace tumor cell origin and fates, and identify drug candidates. In the past decades, several mouse models were employed to study Rb tumorigenesis such as pathological changes and Rb outcomes. However, as a monogenic disease in human, mouse Rb originates from simultaneous mutations in the *RB1* gene and family member *p107* and *p130*. And it has been reported that the occurrence of mouse Rb originates from multiple cell sources as horizontal cell (43), Müller glia cell (44), or amacrine (45), which is drastically different from that of human Rb originating from only cone photoreceptors.

Given the huge gaps between animal models and human Rb, we and other groups have successfully generated human Rb organoid models from PSCs with *RB1*-specific mutations identified in patients. CRISPR/Cas9 gene editing technology was used to construct the *RB1* gene homozygous knockout/mutational hESCs, and Rb tumorigenesis occurred in both cell lines with the directed differentiation of hROs. The Rb model showed a high fidelity of consistency with primary tumor at cellular, transcriptional, and epigenetic modification levels. Besides, we confirmed that maturing cone precursors are the origin of Rb through single-cell sequencing and other approaches. These insights have focused on the development of Rb and the transition and conversion of tumor cells.

However, without patient genetic backgrounds, hESCs-derived Rb organoids cannot completely simulate patient primary tumor because of genetic divergence, which may result in poor guidance in precision medicine. In order to overcome this gap, in this study, we established hiPSCs with an *RB1* heterozygous germline mutation from an Rb patient diagnosed postnatal bilateral retinoblastoma at 10 months old. Sampling tumor tissues could cause Rb intraocular metastases and was therefore not performed in the patient. As a result, we were unable to verify that the tumor was caused by the somatic second hit in the Rb patient. However, Rb patients with germline mutation have a 95% chance of mutating the second allele of the *RB1* gene in at least one susceptible retinal cell in each eye (16). It can be assumed that this was the case in this patient. Nevertheless, we created the second “hit” of the extant functional allele of *RB1* by CRISPR/Cas9 technology. Under differentiation, RBOs were successfully generated from compound heterozygous hiPSCs. Rb organoids were consistent with Rb in transcriptome and protein expression: (1) the gene enrichment of Rb organoids found by RNA-seq analysis were highly related to cell cycle and proliferation; (2) different from *RB1^m1/^^wt^* and *RB1^wt/^^wt^* hROs, Rb-related markers SYK and DEK were highly expressed in both the solid tumor and the adjacent tissues in *RB1^m1/m2^*hRBOs; (3) photoreceptor and precursor markers, CRX and OTX2, were highly expressed in *RB1^m1/m2^* hRBOs, in line with Rb derivation from cone-committed cells; and (4) ARR3^+^ maturing cone precursors are colocalized with proliferative markers Ki67 and PCNA in *RB1^m1/m2^* hRBOs, indicating that Rb is originated from maturing cone precursors. These in vitro organoids recapitulate in accordance with the occurrence and development of Rb tumors, which provides a platform to study tumorigenesis and develop therapeutics for Rb patient.

It is important to note that in *RB1^m1/m2^* hRBOs, we found that the tumor primary foci appeared as early as DD42 during hROs development, which is earlier than the neoplasm formation at DD60 to DD75 in hROs derived from *RB1*-depleted hESCs. This discrepancy may be explained by the patient-specific genetic background. The second explanation is that this may be due to the asynchrony of ROs development from various hESCs or hiPSCs. The last possibility is that the extant functions of mutational Rb alleles are varying. One of the mutations in this study is a frame-shift (p.M208fs) far from C-terminus that could lead to a complete loss of function, and frameshift mutation is typically associated with complete penetrance (46), thus making phenotype more severe. The etiology of Rb is dependent on both genetic and epigenetic factors, which make Rb a multifactorial disorder. And given this, the construction of patient-specific Rb organoids harboring Rb related genetic information is the paramount tool for studying the disease.

Rb has been considered an ideal tumorigenesis model since Knudson proposed the two-hit hypothesis in 1971 (2, 47). One allele of the *RB1* gene was mutated constitutionally or somatically, and the most frequent mutation events are single base substitution, short deletion, or large deletion (48, 49). The other *RB1* gene allele was inactivated somatically due to the loss of heterozygosity, mitotic recombination, promoter hypermethylation, a second base substitution, or others (8, 50–52). Inactivation of both copies of the *RB1* gene is essential for oncogenesis, and one normal allele of the *RB1* gene is protective against tumor development (4, 53). In this study, Rb patient-derived iPSCs carrying heterozygous mutation in the *RB1* gene failed to develop visible tumors during retinal differentiation in dishes. While, after an independent mutation in the other allele of the *RB1* gene, hiPSCs carrying compound heterozygous mutations consistently recapitulated Rb tumorigenesis during retinogenesis in vitro. Our findings have strongly proved that inactivation of both copies of the *RB1* gene is essential for tumorigenesis in retinal organoids, which supports the “two-hit” hypothesis in a straightforward approach.

In 2008, Dimaras et al. suggested that two mutations (M1 and M2) were not sufficient to cause malignant transformation, but to the benign lesion retinoma. Other mutational events such as genomic instability or gains of oncogenes were identified in Rb solid tumor (15). And they proposed that other events (M3-Mn) in retinoma cells are necessary for the progression to malignancy (16, 54, 55). The genomic instability may be a secondary event of *RB1* mutation, as several studies have shown that loss of *RB1* function results in centromere dysfunction and mitotic defects (56–58). Other group and our previous studies have observed retinoma and Rb after *RB1* inactivation, and support the previous assumption that *RB1* depletion may initiate retinoma and subsequently progress to malignancy (31, 41). In this study, we also observed these phenomenon. In summary, we have given strong evidence that the two hits to *RB1* alleles are sufficient for Rb tumorigenesis, which provides a good fit to Knudson's hypothesis.

Xu and colleagues found that *RB1* depletion induces the proliferation of cone precursors rather than other retinal cells (42). And these cone precursors underwent uncontrolled cell division to form tumors, which resemble Rb in structure and function. Singh et al. indicated that the cell-signaling circuitry of human maturing cone precursors is sensitive to *RB1* loss. And in the absence of *RB1*, human maturing (ARR3^+^) cone precursors spontaneously form proliferative lesion with Rb-like histology and protein expression (41). Our data indicate that the tumor cells in Rb organoids expressed ARR3 and Ki67, consistent with the cone origin of human retinoblastoma (41, 42, 59–61).

According to Knudson's “two-hit” hypothesis, both copies deletion of *RB1* are critical for retinoblastoma formation (2). One copy of a certain gene is not enough to maintain normal cellular function, a phenomenon called “haploinsufficiency” (62). In this study, heterozygous *RB1* mutation displayed pathological characteristics in retinogenesis, such as focal neural retinal (NR) dysplasia. In *Rb^+/^^−^; p107^−^^/^^−^* mice model, Lee et al. found the focal lesion in the retina with photoreceptor layer depletion and retinal dysplasia, but did not develop retinoblastoma (63, 64). As confirmed by EM analysis, there were no preneoplastic retinoblastoma cells in focal dysplastic lesions (64). In this work, we offered a *RB1* heterozygous human retina model and proved that loss of one copy of *RB1* is developmentally relevant. Therefore, the loss of one functional *RB1* allele is haploinsufficient in the human retina as well.

In conclusion, patient-specific Rb organoids recapitulate pathological features of human Rb and unzip molecular pathological mechanisms. Both copies of *RB1* are essential for normal retinal cell development; however, the *RB1* haploinsufficiency could not induce Rb tumorigenesis. And most importantly, our findings provide straightforward evidence supporting Knudson's theory.

## Materials and Methods

### Ethical statement

This study was approved by the Institutional Ethical Committee and conducted in accordance with the Declaration of Helsinki. As the patient was a child (13 months), the written informed consent was obtained from his parents. Patient urine sample was collected after the written informed consent was acquired.

### Isolation and expansion of patient urine cells

Urine cells were isolated and cultured as previously reported with minor modifications (65). Six-well plates were precoated with 0.1% (wt/vol) gelatin (Millipore, ES-006-B). The amount of 50 to 200 midstream fresh urine was collected in a sterile bottle. The urine sample was transferred to sterile a 50 mL tube and centrifuged at 400 *g* for 10 min at room temperature (RT), as soon as possible. The supernatant was aspirated carefully and only 1 mL of urine was left in the tube. Urine cells were resuspended and transferred into a sterile 15 mL tube. Prewarmed washing buffer (DPBS containing 100 U/mL penicillin, 100 µg/mL streptomycin, and 2.5 µg/mL amphotericin B) was added up to 15 mL and centrifuged for 10 min at 200 *g* at RT. The supernatant was carefully aspirated and only 0.2 mL of sample was left in the tube. The precipitate containing the urine cells was resuspended with 1 mL of primary medium [45% DMEM/high glucose, 45% Ham's F12 nutrient mix, 10% fetal bovine serum, 1% penicillin–streptomycin, 2.5 µg/mL amphotericin B, and the REGM SingleQuot Kit supplements (Lonza, CC-4127)] and transferred to the gelatin-coated plate. The plate was placed in a 37°C cell incubator with 5% CO_2_ for 24 hours. An amount of 1 mL of primary medium was added daily. After 3 days, the medium was discarded but 1 mL was left, and 1 mL of proliferation medium (RE cell basal medium supplemented with the REGM BulletKit supplements, Lonza, CC-3190) was added. The urine cells were further expanded in proliferation medium with daily exchange of half of the medium. When the urine cells had expanded enough to achieve 80% to 90% confluency in six-well plates, cells were dissociated with TrypLE Select (Life Technology, 12563–011) for further passaging and expansion in proliferation medium. There is an enough number of urine cells for reprogramming at passage 1 or 2.

### Induction and expansion of iPSCs from urine cells

For reprogramming, about 10^5^ to 10^6^ urine cells were dissociated with TrypLE Select and electroporated with 2 µg episomal reprogramming plasmids (System Biosciences, SC900A-1) using LONZA 4D-Nucleofector device (program EA-104). Electroporated urine cells were seeded into six-well plates precoated with Matrigel (0.013 mg/cm^2^) (Corning, 356231) and cultured in 2 mL of Essential 8 (E8) (STEMCELL Technologies, 05990) medium with 10 µM Y-27632 (Selleck, S1049). The plate was incubated in 37°C cell incubator with 5% CO_2_, and the medium was replaced daily. From the second to fourth week after transfection, ESC-like colonies appeared and were picked up and transferred to six-well plates coated with Matrigel. Each colony was expanded as individual iPSC lines.

The hiPSC lines were cultured with the ncEpic hPSC Medium (Nuwacell Biotechnologies Co., Ltd, China, Cat^#^RP01001) in Matrigel-coated six-well plates. The medium as refreshed daily until hiPSCs were expanded to reach above 70% to 80% confluence (around 3 to 5 days after plating). For passaging, cells were dissociated with EDTA and inoculated at a ratio of 1:6.

### CRISPR/Cas9-mediated genome editing

CRISPR/Cas9 technology was used for generating the hiPSCs-*RB1^m1/m2^* with compound heterozygous mutations in the *RB1* gene. The plasmid and targeting vector were generated as described in Liu et al. (31). For genome editing, the accutase (stemcell technologies, 07920) was used for dissociating hiPSCs-*RB1^mut/^^wt^* aggregates into single cells. After centrifuged, 1 × 10^6^ cells were resuspended in the nucleofector solution (Lonza, V4XP-3032) added with 2.5 µg of guide RNA (cDNA)-carrying plasmids and 2.5 µg of *RB1* targeting vector. Cells were electroporated under LONZA 4D-Nucleofector device with the program CA-137. When the nucleofected cells were cultured and reached 70% confluence, puromycin (Gene Operation, ISY1130-0025MG) was added to ncEpic hPSC Medium at a final concentration of 2 µg/mL. A week later, the puro-insensitive colonies were isolated and expanded for genotyping. The RNA and cDNA of each colony were obtained according to the manufacturer's instructions (Fastagne, 220011). And the positive hiPSCs-*RB1^mut/^^mut^* colonies were verified by Sanger Sequencing with the primers described in Liu et al. The online off-target effect assessment software (http://crispr.mit.edu) created by Zhang Feng's Lab was used to find the top 10 potential off-target sites. Genomic DNA was extracted from hiPSC-*RB1^m1/^^wt^* and hiPSC-*RB1^m1/m2^* according to the manufacturer's instruction (Tiangen, DP304), and we amplified the regions that were predicted to have off-target events. Sanger sequencing results showed that there are no mutations caused by the previous gene editing (Fig. S5). The primers used for PCR amplification were listed in Table S1 in the Supplementary Material.

### AP staining

Human iPSCs were cultured in Matrigel coated six-well plates for 3 days. After washing with DPBS, the cells were fixed with 4% paraformaldehyde (PFA) for 15 min at RT. And BCIP/NBT AP Color Development Kit (Beyotime, C3206, China) was used for AP staining according to the manufacturer's protocol. After staining, the cells were washed with DPBS and imaged.

### Three germ layer differentiation of human iPSCs

The hiPSCs were dissociated with 0.5 µM EDTA for 5 min at RT. Incompletely dissociated cell clumps containing 10 to 20 cells were selected and transferred to low attachment plates for further culture in DMEM/F12 supplemented with 0.1 mM NEAA, 0.1 mM Glutamax, 0.1 mM 2-mercaptoethanol, and 1% penicillin–streptomycin. The medium was refreshed every other day until day 9, the cell clumps were transferred to gelatin-coated 24-well plates in DMEM/F12 supplemented with 5% FBS, 0.1 mM NEAA, 0.1 mM Glutamax, 0.1 mM 2-mercaptoethanol, and 1% penicillin–streptomycin. The medium was changed daily, and immunostaining of each germ layer was performed after 10 days of culture.

### Derivation of retinal organoids from hiPSCs

hRO formation was established as previously described with slight modification (26, 27, 36). When hiPSCs were grown to 80% confluence, the TrypLE Select supplemented with 20 µM Y-27632 and 0.05 mg/mL Dnase I was used to dissociate hiPSCs into single cells. About 10,000 to 12,000 cells were plated in each well of the 96-well low-attachment V-bottom plates. EBs were formed in retinal differentiation medium I: GMEM (Gibco) supplemented with 20% KnockOut Serum Replacement (KSR), 1 mM nonessential amino acid, 1 mM sodium pyruvate, 100 µM 2-mercaptoethanol, 1% penicillin–streptomycin (Gibco, 15140163), 20 µM Y-27632, and 3 µM IWR1e. After 48 hours, 2% Matrigel was added to the medium. On the sixth day of differentiation, half of the media was replaced with retinal differentiation medium I without Y-27632. On day 12, the EBs were transferred to 10 cm nonadherent dishes for further culture in retinal differentiation medium II: GMEM supplemented with 10% FBS, 1 mM nonessential amino acid, 1 mM sodium pyruvate, 100 µM beta-mercaptoethanol, 1% penicillin–streptomycin, 1% Matrigel, and 100 nM SAG. On day 18, the transparent neuroepithelium of EBs were manually cut off under microscope, and the medium was exchanged for NR differentiation medium: DMEM/F12 supplemented with 10% FBS, N2 supplement, 0.5 mM retinoic acid, and 0.1 mM Taurine. The NR differentiation medium was changed every 5 to 7 days.

### Immunofluorescence staining

Retinal organoids were fixed in 4% PFA for 1 hour at 4°C, and embedded in OCT Compound (Sakura, 4583). Followed by forzen in liquid nitrogen or dry ice, the embedded organoids were then cut into 12 to 14 µm sections on slides. After washing three times with PBS to remove the OCT, the sections were blocked and penetrated in PBS with 4% bovine serum albumin (Beyotime, ST023) and 0.5% Triton X-100 (Sangon, 600198–0500) for 1 hour at RT. Then sections were washed with PBS, and stained with primary antibodies for 6 to 8 hours or overnight at 4°C. Primary antibodies and their working dilutions were listed in Table S2 in the Supplementary Material. After staining, the sections were rinsed three times with PBS and incubated with secondary antibodies for 1 hour at RT in the dark. Then the slides were washed once with PBS and DAPI was added for nuclei staining. Finally, free antibodies and DAPI were washed away with PBS three times. The slides were sealed with coverslips and stored in the dark. All the images were taken with Olympus (SpinSR10).

### Flow cytometry analysis of cell cycle

Cell cycle analysis was performed according to the manufacturer's instructions (Multi Sciences, Cell Cycle Staining Kit, CCS012). hiPSCs were detached and dissociated into single cells with TryPle Select containing 0.05 mg/mL Dnase I. The cells were washed with DPBS and fixed in cold 75% ethanol overnight at 4°C. The next day, the cells were centrifuged to remove ethanol and resuspended in 2 to 5 mL DPBS and placed at RT for 15 min to fully hydrate. After centrifuged, 1 mL DNA Staining Solution was added and cells were stained for 30 min in the dark. Approximately 10^6^ cells were analyzed using acoustic focusing cytometer (life technologies, Attune NxT). The ModFit 5.0 (Verity Software House) was used for analyzing the percentage of cells in each cell cycle phase.

### Karyotyping of hiPSC cell lines

The karyotypes of hiPSC lines were analyzed by G-banding method (GTG staining) at Nuwacell Biotechnology Co., Ltd. Using proliferating iPSCs, 30 metaphase spreads were counted at minimal resolution of 400 bands. Briefly, iPSCs at passage 31 or 36 were treated with 50 ng/mL colchica at 37°C for 2 hours, trypsinized into single cells, resuspended, and washed once in DPBS. The cells were resuspended in low permeability of KCl at 37°C for 20 to 40 min, fixed with methanol and acetic acid (3:1) overnight. The supernatant was discarded and the cells were dropped onto the precooled slides immediately and dried for 1 to 2 hours at 75°C. The cells on slides were treated with the Giemsa staining and dried at RT. The cells were scanned by Leica scanner (GSL120) and then subjected to chromosome karyotype analysis.

### Micro-CT imaging of retinal organoids

The Zeiss Xradia 515 Versa X-ray microscope (Carl Zeiss AG, Germany) was used to perform high-resolution X-ray CT scanning. To prepare retinal organoid specimen for CT scanning, retinal organoids were pretreated with iodide-potassium iodide (5% iodine in 2% potassium iodide) for 1 hour at RT. After washing three times with DPBS, retinal organoids were loaded in 200 µL pipet tips in 70% ethanol, sealed with paraffin oil and paradilm and attached on tips. Each retinal organoid was scanned individually. The X-ray source was operated at a voltage of 60 kV and a power of 5 W during the scanning process. The CCD optical objective (4X) was used for high resolution images acquisition. The scanning resolution was set to 1.8 µm per vortex under a binding mode of 1.8 × 1.8 for *RB1^wt/^^w^*^t^, 1.5 µm per vortex under a binding mode of 1.5 × 1.5 for *RB1^m1/^^w^*^t^, and 1.2 µm per vortex under a binding mode of 1.2 × 1.2 for *RB1^m1/m2^*. Regardless of the vortex resolution, the thickness of the sample and the internal pore distribution can be clearly seen. Subsequently, 3D reconstruction of obtained images and segmentation for each specimen was performed using Dragonfly software (version 2021). The porosity rate was also calculated using Dragonfly software (version 2021).

### RNA-seq and functional enrichment analysis

An amount of 2 µg total RNA from each sample was used and mRNA was subsequently purified from total RNA using poly-T oligo-attached magnetic beads. RNA sequencing libraries were established by using NEBNext Ultra RNA Library Prep Kit for Illumina (#E7530L, NEB, USA) as recommended. Then the libraries were sequenced on Illumina platform in duplicate or triplicate and 150 base pair (bp) paired-end reads were generated. All reads were mapped to human genome version hg38 by Hisat2 version 7.5.0 with default setting. FeatureCounts (FC) was used for calculating read counts. EdgeR was used for DEG (Log_2_ FC1/FC2 > 1, *P* < 0.05) analysis. The web-based tool Metascape (https://metascape.org/gp/index.html#/main/step1) was used for function enrichment analysis of DEGs of RNA-seq.

### Data collection, visualization, and statistical analyses

The data collection, visualization, and statistical analyses of this project were carried out in the R programming environment. Most analyses were performed with the default settings of software packages unless specified otherwise in the context.

## Authors' Contributions

Z.-B.J. designed and supervised the whole study. Y.-P.L. and Y.-T.W. performed the experiments. W.W. performed the bioinformatic analysis. R.-J.S. performed the sequencing experiments. Y.-P.L. and X.Z. analyzed the data. Y.-P.L. wrote the manuscript. K.J. and Z.-B.J. critically reviewed and revised the manuscript.

## Supplementary Material

pgac162_Supplemental_Files

## Data Availability

RNA-seq data have been deposited in the National Genomics Data Center (NGDC), part of the China National Center for Bioinformation Center (accession no. HRA002657).
