# Supplementary material for: Second hit impels oncogenesis of retinoblastoma in patient-induced pluripotent stem cell-derived retinal organoids: direct evidence for Knudson's theory
Source: PNAS Nexus. 2022 Aug 17;1(4):pgac162. doi: 10.1093/pnasnexus/pgac162 (PMC9802398; doi:10.1093/pnasnexus/pgac162)
Supplement: pgac162_Supplemental_Files [file pgac162_supplemental_files.zip › PNASNEXUS-PNASNEXUS-2022-00487-s01.docx]

**
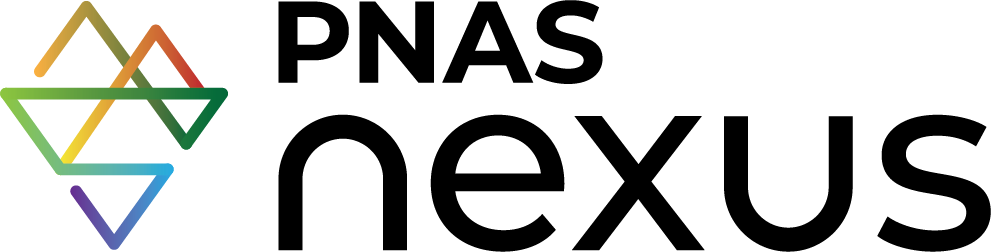
**

**Supplementary Information for**

Second Hit Impels Oncogenesis of Retinoblastoma in Patient-iPSC-derived Retinal Organoids: Direct Evidence for Knudson’s Theory

Yan-Ping Li^1^, Ya-Ting Wang^1^, Wen Wang^1^, Xiao Zhang^1^, Ren-Juan Shen^1^, Kangxin Jin^1^, Li-Wen Jin^2^, Zi-Bing Jin^1*^

1. Beijing Institute of Ophthalmology, Beijing Tongren Hospital, Capital Medical University, Beijing 100730 China;

2. Quanzhou Aier Eye Hospital, Quanzhou, 362017, China.

Correspondence: Dr. Zi-Bing Jin, Beijing Institute of Ophthalmology, Beijing Tongren Hospital, Capital Medical University, Beijing 100730 China.

E-mail: jinzibing@foxmail.com

**This PDF file includes:**

Supplementary text

Figures S1 to S5

Tables S1 to S2

Legends for Movies S1 to S4

**Other supplementary materials for this manuscript include the following:**

Movies S1 to S4

**
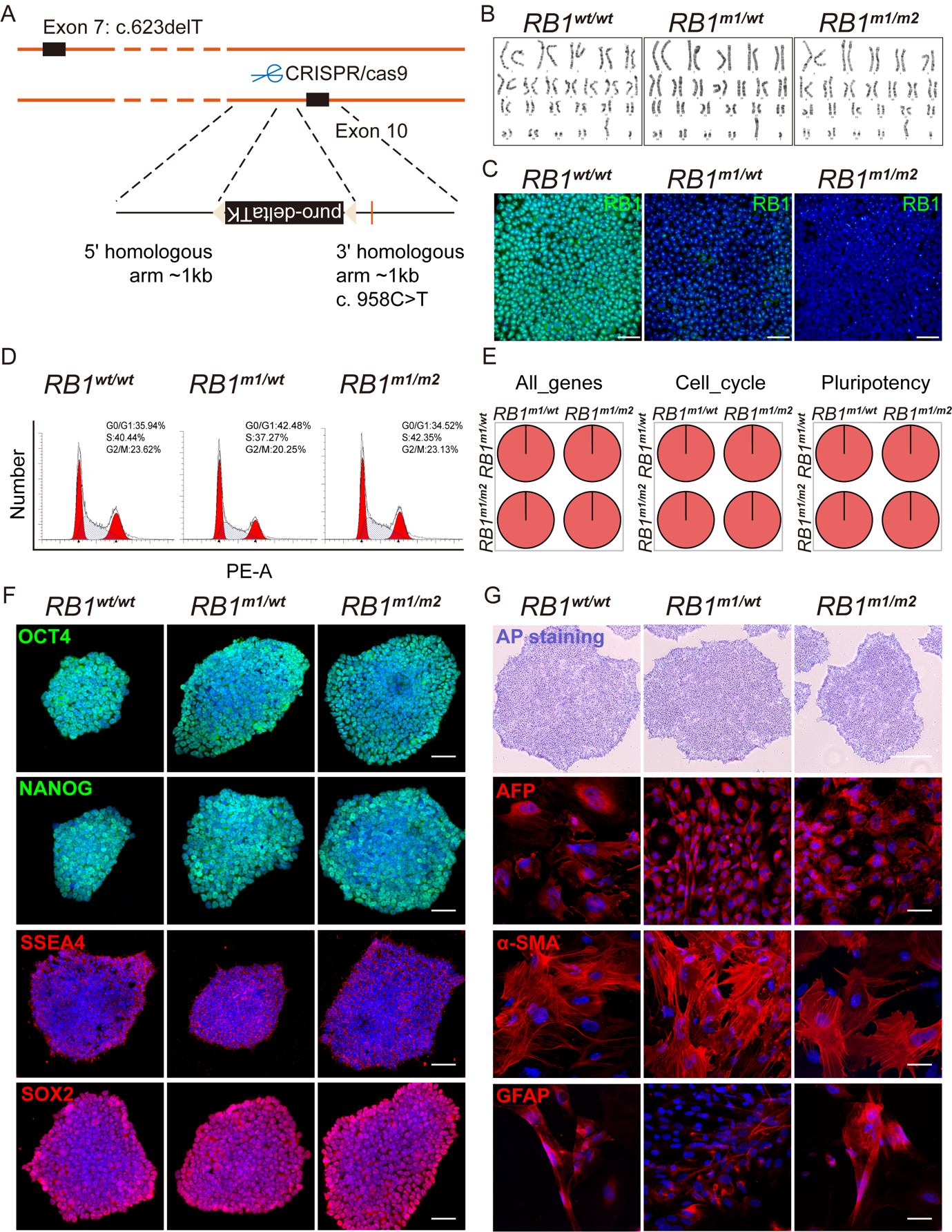
Supplementary Information Text**

**Figure S1. Characterization of hiPSC-*RB1^wt/wt^*, -*RB1^m1/wt^* and -*RB1^m1/m2^* cell lines. (A)** Schematic diagram of point mutation knock-in targeting *RB1* allele for the construction of compound heterozygous hiPSC line using Crispr/cas9. **(B)** Verification of karyotypes in iPSC-*RB1^wt/wt^*, -*RB1^m1/wt^* and -*RB1^m1/m2^* lines. **(C)** Representative images of immunostaining for RB1 protein expression in iPSC-*RB1^wt/wt^*, -*RB1^m1/wt^* and -*RB1^m1/m2^* lines. Scale bars, 50 µm. **(D)** Cell cycle analysis of iPSC-*RB1^wt/wt^*, -*RB1^m1/wt^* and -*RB1^m1/m2^* lines. **(E)** Correlation analysis of the expression of all genes, cell cycle and pluripotency associated genes between iPSC-*RB1^m1/wt^* and iPSC-*RB1^m1/m2^* based on RNA-seq data. **(F)** Immunostaining for OCT4, NANOG, SSEA4, and SOX2 expression in iPSC-*RB1^wt/wt^*, -*RB1^m1/wt^* and -*RB1^m1/m2^* lines. Scale bars, 50 µm. **(G)** Representative images of AP staining and immunostaining for AFP, α-SMA, GFAP in iPSC-*RB1^wt/wt^*, -*RB1^m1/wt^* and -*RB1^m1/m2^* lines. Scale bars, 400 µm in AP staining, 50 µm in immunostaining. Cell nucleus was counterstained with DAPI (blue) in all **
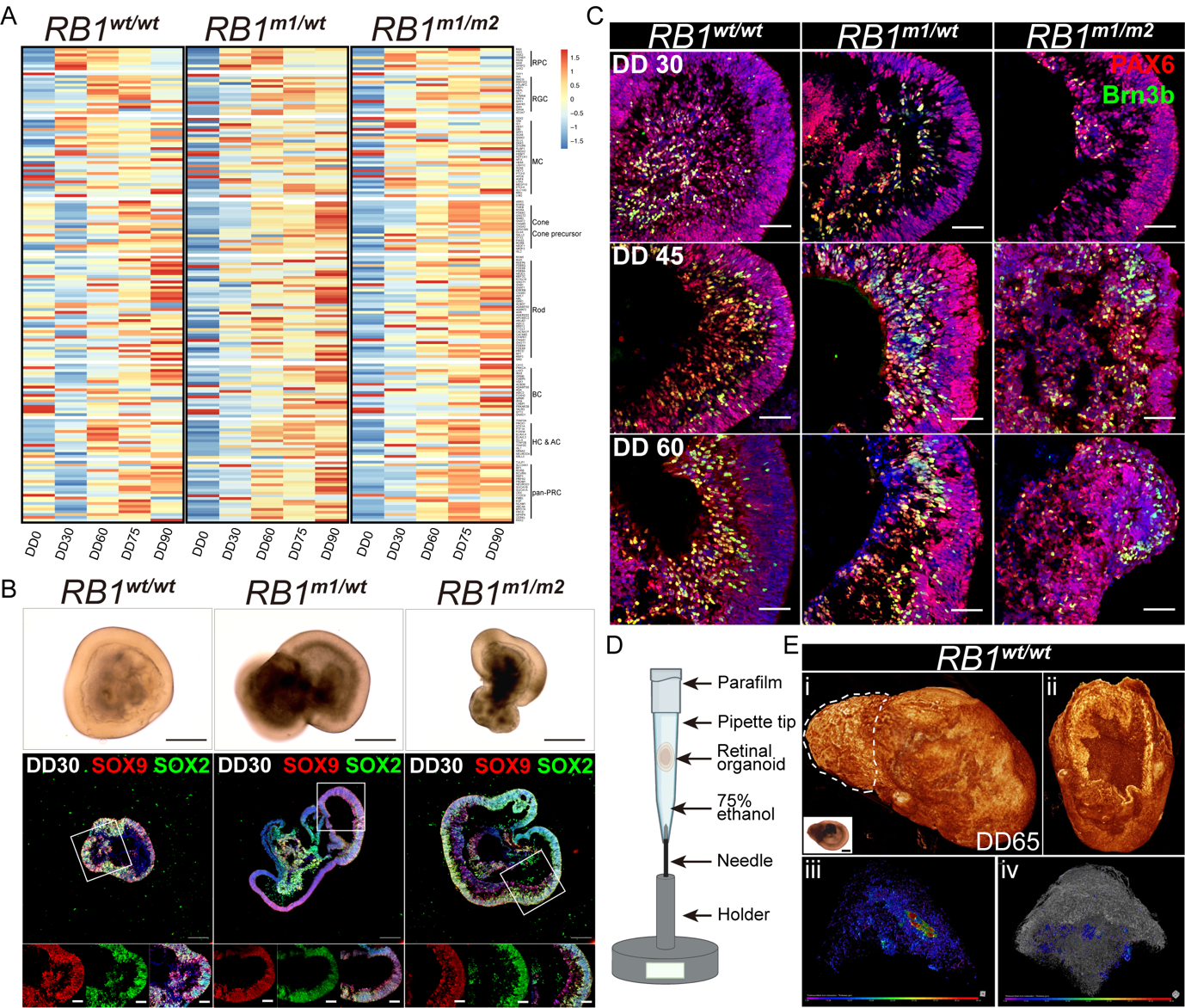
**immunostaining images (C, F and G, but not in AP staining).

**Figure S2. Morphological and molecular characterization of *RB1^wt/wt^*, *RB1^m1/wt^* and *RB1^m1/m2^* hROs. (A)** Heatmap showed the clustering of gene expression at various stages of retinal cell genesis in *RB1^wt/wt^*, *RB1^m1/wt^* and *RB1^m1/m2^* hROs. **(B)** Representative images of *RB1^wt/wt^*, *RB1^m1/wt^* and *RB1^m1/m2^* hROs and their expression of early proliferative markers SOX2 and SOX9 at DD30. Top, bright field images of retinal organoids; scale bars, 400 µm. Middle, hROs at DD30 were immunostained with SOX2 (green) and SOX9 (red) and counterstained with DAPI (blue); scale bars, 200 µm. The boxed regions were magnified in the bottom as single channel or merged images; scale bars, 50 µm. **(C)** Immunostaining of PAX6, and POU4f2 (Brn3b) in ROs at DD30, 45, and 60. Cell nucleus was counterstained with DAPI (blue). Scale bars, 50 µm. **(D)** Schematic of organoids holding for microCT imaging. **(E)** The microCT imaging of organoids. (i), The reconstructed 3D images of the *RB1^wt/wt^* hRO at DD65 scanned with 1.8 µm vortex resolution; bright-filed image of *RB1^wt/wt^* hRO was shown in the bottom left corner, Scale bars, 400 µm. (ii), The cross-section of 3D rendering of *RB1^wt/wt^* hRBO. (iii and iv), 3D rendering images were obtained from Dragonfly **
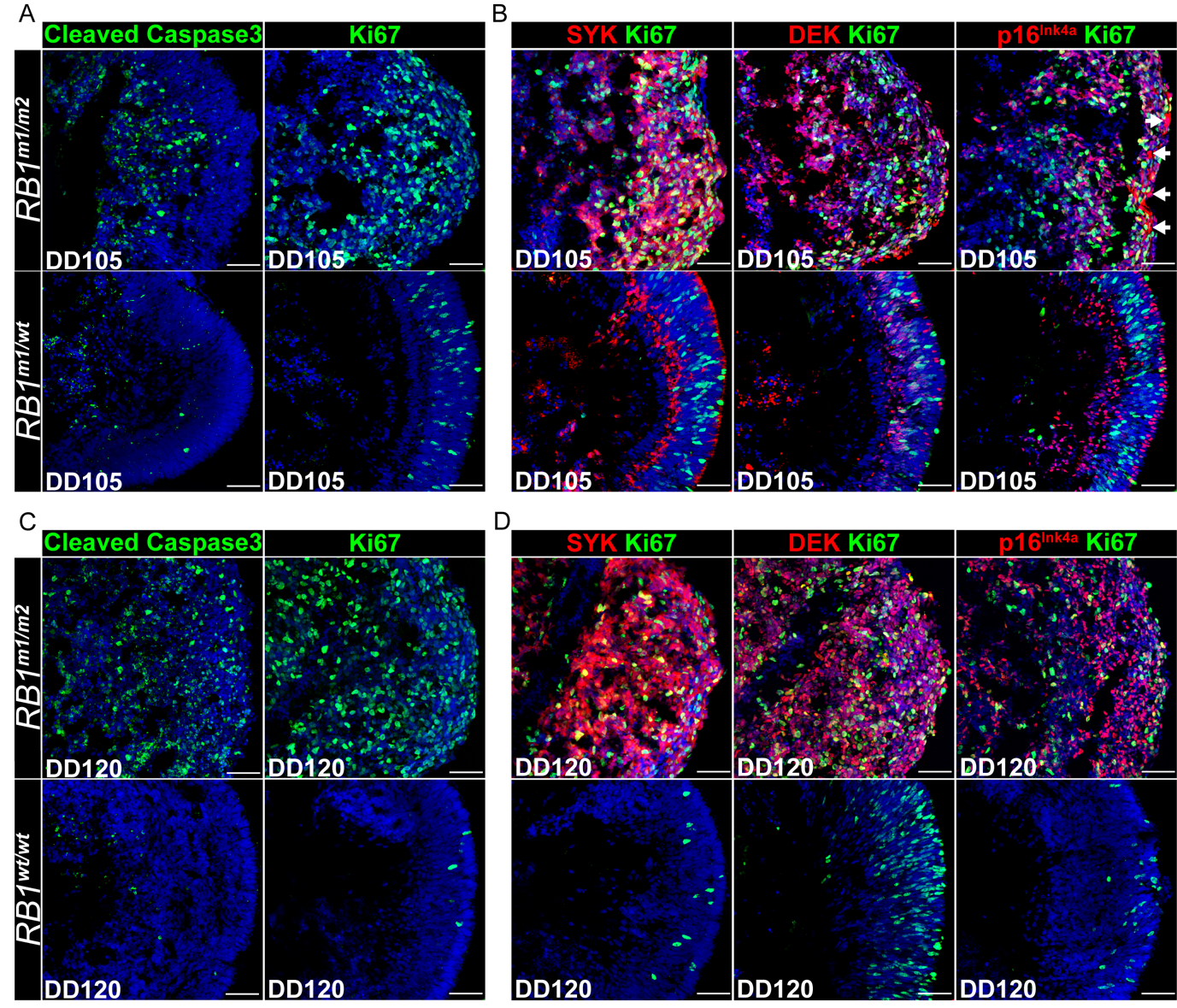
**software’s thickness analysis of the organoid’s internal pores and surface.

**Figure S3. Expression of cell apoptosis, proliferation and tumor-related markers in *RB1^m1/wt^* and *RB1^m1/m2^* hROs at DD105 and 120.** **(A)** Immunostaining of Cleaved Caspase 3 and Ki67 in *RB1^m1/wt^* and *RB1^m1/m2^* hROs at DD105. Scale bars, 50 µm. **(B)** Immunostaining of SYK, DEK and p16^Ink4a^ in *RB1^m1/wt^* and *RB1^m1/m2^* hROs at DD105. White arrows pointed to representative cells with high cytoplasmic p16^Ink4a^ in hRBOs. Scale bars, 50 µm. **(C)** Immunostaining of Cleaved Caspase 3 and Ki67 in *RB1^wt/wt^* and *RB1^m1/m2^* hROs at DD120. Scale bars, 50 µm. **(D)** Immunostaining of SYK, DEK and p16^Ink4a^ in *RB1^m1/m2^* and *RB1^wt/wt^* hROs at DD120. Scale bars, 50 µm. Cell nucleus was counterstained with DAPI in all **
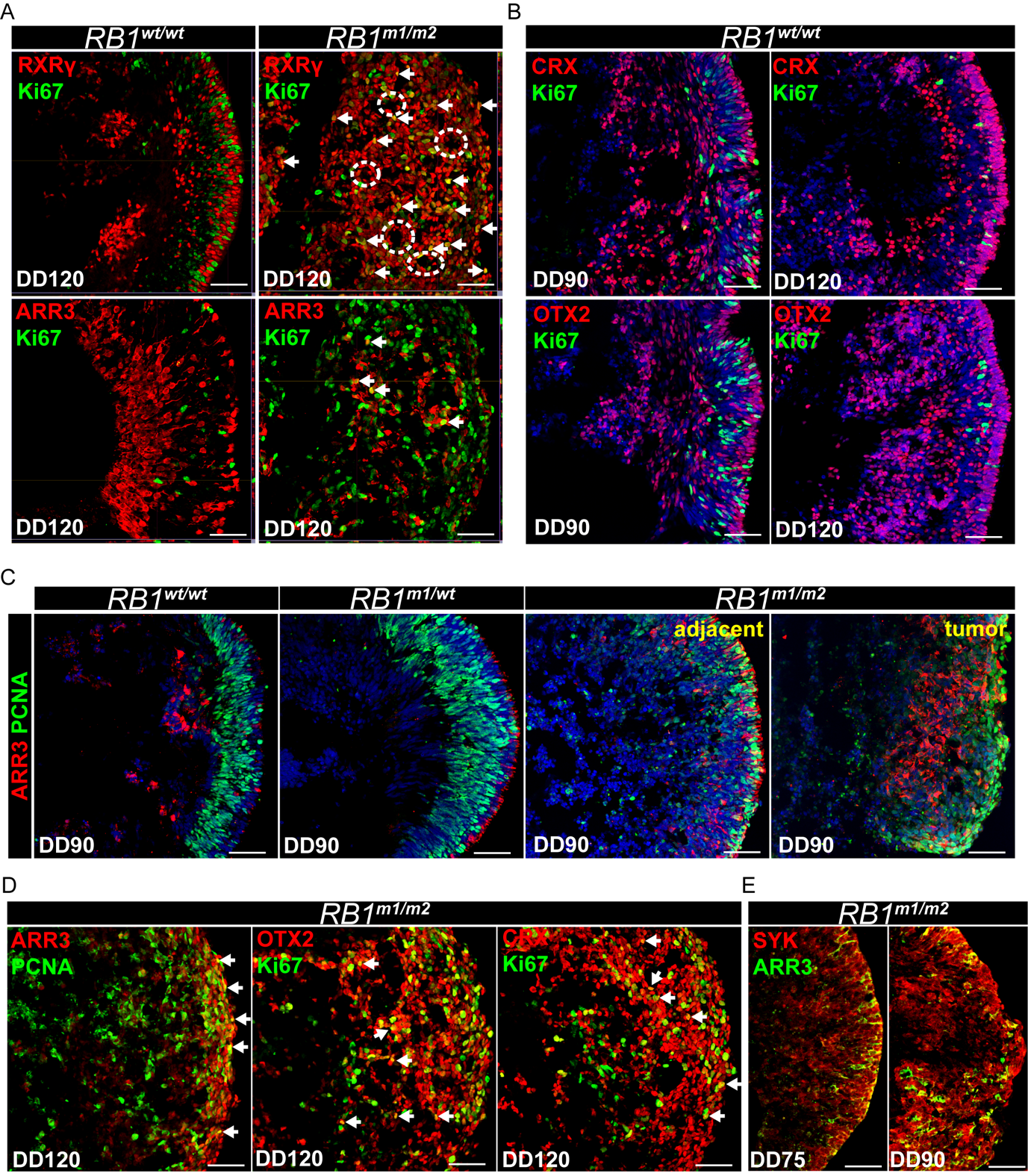
**images.

**Figure S4. Cells of tumor and adjacent tissues in *RB1^m1/m2^* hRBOs express cone and cone precursor markers.** **(A)** Immunostaining of Ki67 and cone precursor marker RXRγ and maturing cone marker ARR3 in *RB1^m1/m2^* hRBOs and *RB1^wt/wt^* hROs at DD120. White arrows pointed to cells with co-expression of Ki67 and RXRγ or ARR3. Dashed circles enclosed the Flexner-Wintersteiner rosette-like structures. Scale bars, 50 µm. **(B)** Immunostaining of photoreceptor-determining transcription factors CRX and OTX2 in *RB1^wt/wt^* hROs at DD90 and 120. Cell nucleus was counterstained with DAPI. Scale bars, 50 µm. **(C)** Immunostaining of ARR3 and PCNA in hROs at DD90. Cell nucleus was counterstained with DAPI. Scale bars, 50 µm. **(D)** Immunostaining of ARR3, PCNA, OTX2, CRX and Ki67 in *RB1^m1/m2^* hRBOs at DD120. White arrows pointed to co-labeled cells. Scale bars, 50 µm. **(E)** Immunostaining of SYK and ARR3 in *RB1^m1/m2^* hRBOs at DD75 and 90. Scale bars, 50 µm.

**
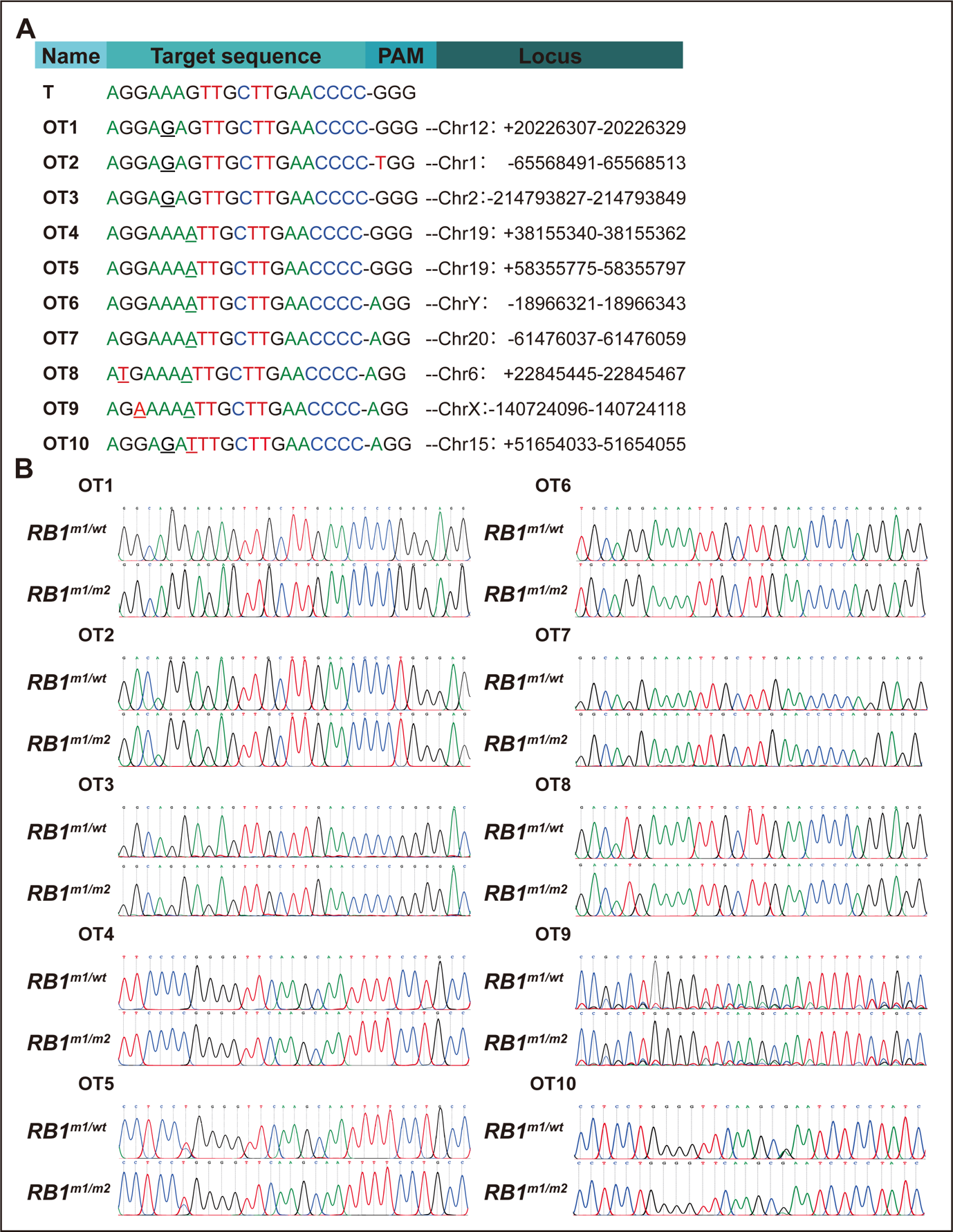
Figure S5. Analysis of off-target editing in top ten candidate sites.** (A) The sequences and positions of top ten possible off-target sites for sgRNA/Cas9. T, target site sequence; OT1～OT10, ten predicted off-target sites and sequences. The mismatch sites are underlined. (B) Sanger sequencing of the ten putative off-target sites with high-fidelity PCR from iPSC-RB1m1/wt and iPSC-RB1m1/m2 genomes found no off-target modifications.

**Table S1. The primers used for PCR amplification of the ten predicted off-target sites.**

| Target site | Primer |
| --- | --- |
| OT1 | Forward GCAGGTGGATCATGAGGTCA  Reverse CGCTCCTGATTTGCCTTTTCTT |
| OT2 | Forward GCATGTGGGCTATGTGATTTCT  Reverse AGACCAGTCCAACCCATACG |
| OT3 | Forward GGCAGGGAGGAGGAGGATAAATAAG  Reverse GATGTTTAAGTGCTGGGTGGTTG |
| OT4 | Forward GTGGTGGGTGCCTGTAGT  Reverse ACCTGTTCCTTCCAAATTTGCT |
| OT5 | Forward AAAATTAGCCGGGCATGGTG  Reverse CATTCATGCTGTCCCTTGGG |
| OT6 | Forward AGTGTCTCTCTCTGTTGCCC  Reverse TCCCAGTGATTTGCAAAGCC |
| OT7 | Forward CATCACGCCTGGCTTTCAAC  Reverse CATGGCGCTTGTGTCCTC |
| OT8 | Forward TGTGGCCAGGAGTTCAAGAT  Reverse CTCTCTCATGCTGTCACCCA |
| OT9 | Forward CCTCACCAGATGCTAACACCA  Reverse GGCAGATCACAAGGTCAGGA |
| OT10 | Forward AAAGTGGTTGGGCGTGATTG  Reverse CCCCTCCCTAATCCTCCTCT |

**Table S2. Primary and secondary antibodies used in this study.**

| Antibodies | Source | Identifier | Dilution ratio |
| --- | --- | --- | --- |
| Anti-Arrestin 3 | Novus Biologicals | Cat# NBP1-37003 | 1:200 |
| Anti-Syk (4D10) | Santa Cruz Biotechnology | Cat# sc-1240 | 1:200 |
| Anti-Ki67 antibody | Abcam | Cat# ab15580 | 1:400 |
| Anti-Ki67 antibody | BD Biosciences | Cat# 550609 | 1:400 |
| Anti-Ki67 antibody | Invitrogen | Cat# 14-5698-82 | 1:100 |
| Anti-RXRγ (A-2) | Santa Cruz Biotechnology | Cat# sc-365252 | 1:200 |
| Anti-CRX (M02) | Abnova | Cat# ABN-H00001406-M02 | 1:400 |
| Anti-OCT4 | Abcam | Cat# ab18976 | 1:200 |
| Anti-SOX-2 (E-4) | Santa Cruz Biotechnology | Cat# sc-365823 | 1:200 |
| Anti-NANOG | Abcam | Cat# ab109250 | 1:200 |
| Anti-SSEA4 | Abcam | Cat# ab16287 | 1:100 |
| Anti-NSE antibody | Novus Biologicals | Cat# NBP2-47635 | 1:200 |
| Anti-DEK antibody | BD Biosciences | Cat# 610948 | 1:400 |
| Anti-CDKN2A/p16INK4a | Abcam | Cat# ab108349 | 1:200 |
| Anti-α-SMA | Abcam | Cat# ab7817 | 1:200 |
| Anti-GFAP | Santa Cruz Biotechnology | Cat# sc-33673 | 1:200 |
| Anti-AFP | R&D systems | Cat# MAB1368-SP | 1:50 |
| Anti-Rb antibody [EPR17512] | Abcam | Cat# ab181616 | 1:800 |
| Anti-RX | Santa Cruz Biotechnology | Cat# sc-271889 | 1:300 |
| Anti-Pax-6 | Biolegend | Cat# 901301 | 1:300 |
| Anti-SOX9 [EPR14335-78] | Abcam | Cat# ab185966 | 1:400 |
| Anti-Brn3b | Novus Biologicals | Cat# NBP2-75098 | 1:50 |
| Anti-PKC-α (H-7) | Santa Cruz Biotechnology | Cat# sc-8393 | 1:200 |
| Anti-Otx2 | Abcam | Cat# ab183951 | 1:200 |
| Anti-Cleaved Caspase3 | Cell Signaling Technology | Cat# 9664S | 1:400 |
| Anti-PCNA (PC10) | Santa Cruz Biotechnology | Cat# sc-56 | 1:200 |
| Donkey anti-Goat IgG (H+L) Secondary Antibody, Alexa Fluor Plus 488 | Thermo Fisher Scientific | Cat# A32814 | 1:400 |
| Alexa Fluor 594 AffiniPure Donkey Anti-Mouse IgG (H+L) | Jackson ImmunoResearch | Cat# 715-585-151 | 1:400 |
| Alexa Fluor® 488 AffiniPure Donkey Anti-Rabbit IgG (H+L) | Jackson ImmunoResearch | Cat# 711-545-152 | 1:400 |
| Donkey anti-Rabbit IgG (H+L) Secondary Antibody, Alexa Fluor 594 | Thermo Fisher Scientific | Cat# A-21207 | 1:400 |
| Donkey anti-Mouse IgG (H+L) Highly Cross-Adsorbed Secondary Antibody, Alexa Fluor 488 | Thermo Fisher Scientific | Cat# A-21202 | 1:400 |
| Goat anti-Rat IgG (H+L) Cross-Adsorbed Secondary Antibody, Alexa Fluor 647 | Thermo Fisher Scientific | Cat# A-21247 | 1:400 |

**Table S2. Antibodies used in this study.**

**Movie legends**

**Movie S1. Abnormal proliferation of *RB1^m1/m2^* iPSC-derived hRBOs (days 56-64).** The live movie in which tumor was visualized to bulged outward (white arrow)and grew rapidly (dashed line). Images every 1 hour for 9 days.

**Movie S2. The microCT movie of *RB1^m1/m2^* iPSC-derived hRBOs (DD 55).** The reconstructed 3D movie of the *RB1^m1/m2^* hRBO scanned with 1.2 µm vortex resolution. 3D rendering movie were obtained from Dragonfly software.

**Movie S3. The microCT movie of *RB1^m1/wt^* iPSC-derived hROs (DD 55).** The reconstructed 3D movie of the *RB1^m1/wt^* hRO scanned with 1.5 µm vortex resolution. 3D rendering movie were obtained from Dragonfly software.

**Movie S4. The reconstructed 3D movie of *RB1^wt/wt^* iPSC-derived hROs (DD 65).** The reconstructed 3D movie of the *RB1^wt/wt^* hRO at DD65 scanned with 1.8 µm vortex resolution. 3D rendering movie were obtained from Dragonfly software.
